# Dr. Charles A. St. John

**Published:** 1901-07

**Authors:** 


Dr. Charles A. St. John, U. of B., '98, was killed in the Province of North Camarines, P.I., May 22, 1901. Dr. St. John was attached to the 26th Infantry as a medical officer. He was reported last October as dead from typhoid fever, but this proved an error, and he afterward performed excellent service. He was on duty in the field with the troops when he lost his life.

